# Research on pragmatic impairment in autistic children during the past two decades (2001–2022): hot spots and frontiers—based on CiteSpace bibliometric analysis

**DOI:** 10.3389/fpsyg.2024.1276001

**Published:** 2024-09-12

**Authors:** Yanqin Liu, Xin Tian, Haoran Mao, Lulu Cheng, Peng Wang, Yang Gao

**Affiliations:** ^1^School of Foreign Studies, China University of Petroleum (East China), Qingdao, China; ^2^Institute of Manchu Studies, Heilongjiang University, Harbin, China; ^3^School of Foreign Languages, Jiamusi University, Jiamusi, China; ^4^Shanghai Center for Research in English Language Education, Shanghai International Studies University, Shanghai, China; ^5^Department of Language, Literature and Communication, Faculty of Humanities, Vrije Universiteit Amsterdam, Amsterdam, Netherlands; ^6^Western Studies of Heilongjiang University, Harbin, China; ^7^School of Foreign Languages, Harbin University of Commerce, Harbin, China

**Keywords:** autistic children, pragmatic impairment, research hotspot, research frontier, theory of mind

## Abstract

Pragmatic impairment has become a critical aspect of language development in autistic children and has gained significant academic attention over the past two decades. This study leverages bibliometric methods to conduct an exhaustive analysis of literature derived from Web of Science database. Utilizing CiteSpace software, we construct a knowledge map to dissect the academic hotspots in research related to pragmatic impairment in autistic children. This enables us to delineate the evolutionary trajectory of this research domain, analyze the prevailing research dimensions, and anticipate potential future dimensions. Our findings indicate that research hotspots in this field over the past two decades predominantly concentrate on assessing and diagnosing pragmatic impairment in autistic children, intervention strategies, and theory of mind. The research scope on pragmatic impairment in autistic children has progressively broadened and deepened. Research has evolved from initial descriptions and interpretations of autism to exploring the theory of mind in high-functioning, school-aged children. The current emphasis is on examining the specific skills that these children possess.

## 1 Introduction

Autism Spectrum Disorder (ASD) is a pervasive neurodevelopmental disorder that influences approximately 1% of the global populace, with a notable upward trend across a broad spectrum of geographic and sociodemographic groups (Zeidan et al., 2022). ASD is primarily characterized by challenges in social communication, constricted interests, and repetitive behaviors, all of which culminate in suboptimal social interaction capabilities for those on the spectrum. It is critical to recognize that ASD, in particular, exhibits a high incidence rate among children. In the United States, approximately 1 in 36 children is affected by ASD (ASD, 2023), whereas in China, according to *the Report on the Development Status of the Autism Education and Rehabilitation Industry in China* (III), the figure has surpassed 10 million, with over 200 thousand increasing every year (Menglin, 2019). Among children on the autism spectrum, their language development is notably impacted from a pragmatic perspective. When viewed through this lens, autistic children often experience various incomplete stages in language processing and usage (Cantiani et al., 2016; DiStefano et al., 2019). Those difficulties, evident in social communication among those with autism, often manifest as verbal or non-verbal challenges. These individuals typically struggle with social conventions and shifting between the roles of speaker and listener in conversations, highlighting the pragmatic complexities of their social interactions. Given the considerable prevalence of these challenges in children and their impact on social communication (Cheng et al., 2022), there has been a heightened emphasis on research efforts to improve the pragmatic skills of autistic children.

In contrast to pragmatically impaired language use, effective pragmatic language encompasses several aspects. This includes the dynamic adaptation of language based on situational factors, such as formality levels (e.g., formal versus informal speech). It also involves a mastery in understanding and employing implied meanings and non-literal language, including idioms, metaphors, irony, and sarcasm.

However, for children on the autistic spectrum, pragmatic impairment stands as a primary obstacle preventing them from effectively participating in social activities. This impairment, notable for its adverse effects, typically manifests as inappropriate pragmatic use of language and is often pinpointed during critical stages of language development in these children (Zhou, 2011). Most of its observable traits are characterized by language behaviors that lack the appropriate pragmatic relevance in a social context, stemming from the deficits associated with ASD. Consequently, investigating pragmatic impairment in autistic children holds paramount significance. Such studies have the potential to enhance their communicative language abilities, with a specific focus on strengthening their social communication skills.

Research on pragmatic impairments among autistic children has rapidly developed and become a trending topic, largely driven by the progressive trend in ASD research and the increasing public understanding witnessed over the past two decades. Researchers typically define ASD using the clinical diagnostic criteria from either the International Classification of Diseases (10th edition) (ICD-10) (World Health Organization, 1992) or the Diagnostic and Statistical Manual of Mental Disorders (5th edition) (DSM-5) (American Psychiatric Association, 2013). Both are widely accepted diagnostic systems for mental disorders. Autistic children often exhibit genetic abnormalities and etiologies that co-occur with brain developmental issues, distinguishing them from neurotypical children (Kempe and Bauman, 1998; Casanova et al., 2002). The DSM-5 offers detailed diagnostic criteria for language impairments in autistic children, including delays or lack of language development, challenges in initiating or maintaining conversation, and tendencies toward restricted, repetitive, or idiosyncratic communication behaviors (American Psychiatric Association, 2013). Early research, informed by this manual, aimed to identify unique language characteristics in autistic children. These studies produced a range of observations, such as non-grammatical word choices, reverse references in personal pronouns, disorganized discourse structures, unresponsive answers to questions, abnormal intonation or prosody, and a lack of communicative intent. However, these observations largely focus on a micro dimension, emphasizing the more subtle linguistic challenges faced by autistic children. However, a more pressing concern that frequently goes unnoticed for these children is their struggle to develop pragmatic competence—a challenge representing the macro dimension. This broader purview encapsulates the holistic spectrum of adversities, ranging from nuanced self-manifestations in discerning communication contexts to the interpretation of non-verbal cues, inferential reasoning, and outcome anticipation (Kotila et al., 2020; Pritzker, 2020).

Prior research consistently shows that, even before the preschool years, language comprehension in autistic children is often impaired, especially when compared to both children with intellectual disabilities (who possess similar nonverbal cognitive abilities) (Bartak et al., 1977) and their neurotypical peers (Tager-Flusberg, 1981). Interestingly, recent findings suggest that low comprehension may be an age-specific marker for autistic children, as their language comprehension tends not to remain significantly impaired beyond the preschool years (Mathée-Scott and Ellis, 2022). The literature provides substantial evidence that pragmatic deficits (pertaining to the use, processing, and production of language in communication) is one of the social communication barriers in preschool-aged children. Scoping at autistic children at preschool ages, they often display a deficiency in speech comprehension and pragmatic production.

Four highly related factors for those deficiencies have been revealed by recent studies. First, deficit of theory of mind (ToM) serves as a contributor to pragmatic impairment. Autistic children may struggle with understanding others’ intentions, inferring the meaning behind speech, and predicting others’ behavior due to deficiencies in ToM. For instance, deficits in ToM may impact autistic children’s ability to understand non-literal or figurative meanings of speech. When we use figurative language, such as “she is a butterfly,” most people can understand that this metaphor means describing someone as beautiful or free as a butterfly. However, for some autistic children, they may interpret this sentence more literally and think that the person being described is actually a butterfly (Baixauli-Fortea et al., 2019). Second, studies employing electrophysiological and imaging methodologies evidenced that language development, including using language effectively in social contexts in autistic children, can also be obstructed by hearing impairment (Klein et al., 1995). Some autistic children might also have co-occurring hearing impairments. Communication difficulties associated with both conditions can lead to misunderstandings, frustration, and social isolation. Those challenges can interact and compound communication difficulties, eliciting or reinforcing pragmatic impairment. In addition, pronounced non-acoustic deficits in speech reception also contribute to an increased risk of epilepsy (Fombonne, 1999). With higher prevalence of epilepsy in individuals with ASD during childhood, they underwent difficulties in accurately perceiving and processing spoken language which directly cause pragmatic impairment. For instance, autistic children in the two conditions might have trouble in distinguishing between different speech sounds (phonemes), recognizing patterns in speech, and understanding the meaning of spoken words, leading to interruptions or difficulty maintaining a back-and-forth exchange in social interaction. Furthermore, genetic studies have brought to light that family members of individuals with ASD bear a higher risk of developing other developmental disorders, such as language disorders (le Couteur et al., 1996). That is, certain traits or conditions are more commonly found within families of ASD compared to the general population. Autistic children who are genetically closer to an individual with ASD in such family are more likely to share some of the same pragmatic challenges. All these factors correlate with the developmental level of their pragmatic abilities.

Therefore, it is of paramount importance to delve into research on the pragmatic impairment of autistic children, investigate its underlying pathological causes, and devise effective scientific interventions. These efforts are critical to reducing the prevalence of ASD and enhancing the pragmatic competence and overall quality of life of autistic children.

Consequently, this study focuses on the literature related to pragmatic impairments in autistic children, sourced from Web of Science database. With the assistance of the CiteSpace visual analysis tool, this study aims to identify the hot topics, central themes, and evolution of autism research from 2001 to 2022. The purpose is to scrutinize the evolution and emerging trends in research on pragmatic impairments in autistic children. This endeavor will not only augment the scientific research landscape in this field, but also yield invaluable insights for future research.

## 2 Research samples and procedures

### 2.1 Data source and searching strategy

Data was extracted from Web of Science Core Collection (WoSCC). A comprehensive keyword search was conducted employing the search strings “autistic children* pragmatic impairment” and “children with autism* pragmatic impairment.” The search parameters encompassed titles, abstracts, author-provided keywords, and extended keywords. To uphold the quality of the incorporated literature, manual screening was executed to exclude non-relevant sources, such as brief news articles and conference reviews. This screening process culminated in the compilation of 255 pertinent literature articles pertaining to pragmatic impairment in autistic children. The data for this study was retrieved on May 17, 2022.

### 2.2 Study procedures

The present study implemented the CiteSpace 5.7.R5 (64-bit), an advanced scientific literature visualization and data processing tool (Chen et al., 2015), to streamline the analysis of the collected valid sample documents. The samples were meticulously exported and subsequently subjected to a process of visualization. This procedure entailed the generation of co-occurrence diagrams, cluster diagrams, emergence analysis diagrams, and timeline diagrams of keywords that have been prominently used in the realm of ASD-related pragmatic impairment research. These visual analytic parameters pave the way for an in-depth exploration of contemporary prominent themes and emerging research trajectories, effectively spotlighting the developmental progression of the discipline.

## 3 Results

The temporal distribution of literature publications presents some significant trends in research concerning pragmatic impairment in ASD spanning the last two decades. The data delineate four conspicuous phases marked by early emergence, gradual progression, swift proliferation, and sustained development. In the period from 2001 to 2007, the annual count of publications did not surpass 5, indicating that the study on pragmatic impairment in autistic children was in its nascent stage. The subsequent period, from 2008 to 2014, witnessed a steady yet modest increment in annual publications, averaging around 5, which underscored a period of slow growth in the unveiling of relevant research findings.

A remarkable surge in publication was observed between 2015 and 2017, with a total of 75 papers published, surpassing the cumulative total of the preceding 14 years. Of particular note is the apex in 2017, with 36 papers published within the year. Post 2018, the research into pragmatic impairment in autistic children transitioned on a phase of variable growth, with 9 publications as of mid-May 2022. It can be reasonably projected that, as the theoretical underpinnings and experimental investigations pertaining to pragmatic impairment in ASD continue to deepen, there will be a considerable augmentation in the volume of published literature, heralding a new epoch of academic growth in this field.

Research on pragmatic impairment in autistic children is a shared endeavor spanning various disciplines, emblematic of significant multidisciplinary collaboration and interdisciplinary convergence. The involving disciplines encompass linguistics, psychology, and clinical medicine. There is a profusion of publications within the realms of linguistics and developmental psychology centered on pragmatic impairment research. Concurrently, the field of clinical medicine, particularly within sub-disciplines such as rehabilitation, pathology, psychiatry, pediatrics, and clinical neurology, has made substantial contributions to ASD-related research.

The top 10 research fields collectively contributed more than 392 publications, surpassing the total of 255 documents collected. This highlights the instances where multiple documents address the same research question yet span across disparate disciplines. Such a phenomenon illustrates the breadth of interdisciplinary synergies operating within the field of pragmatic impairment in ASD research, fostering the generation of insightful research outcomes.

### 3.1 Research hotspots analysis

**Figure 1:**
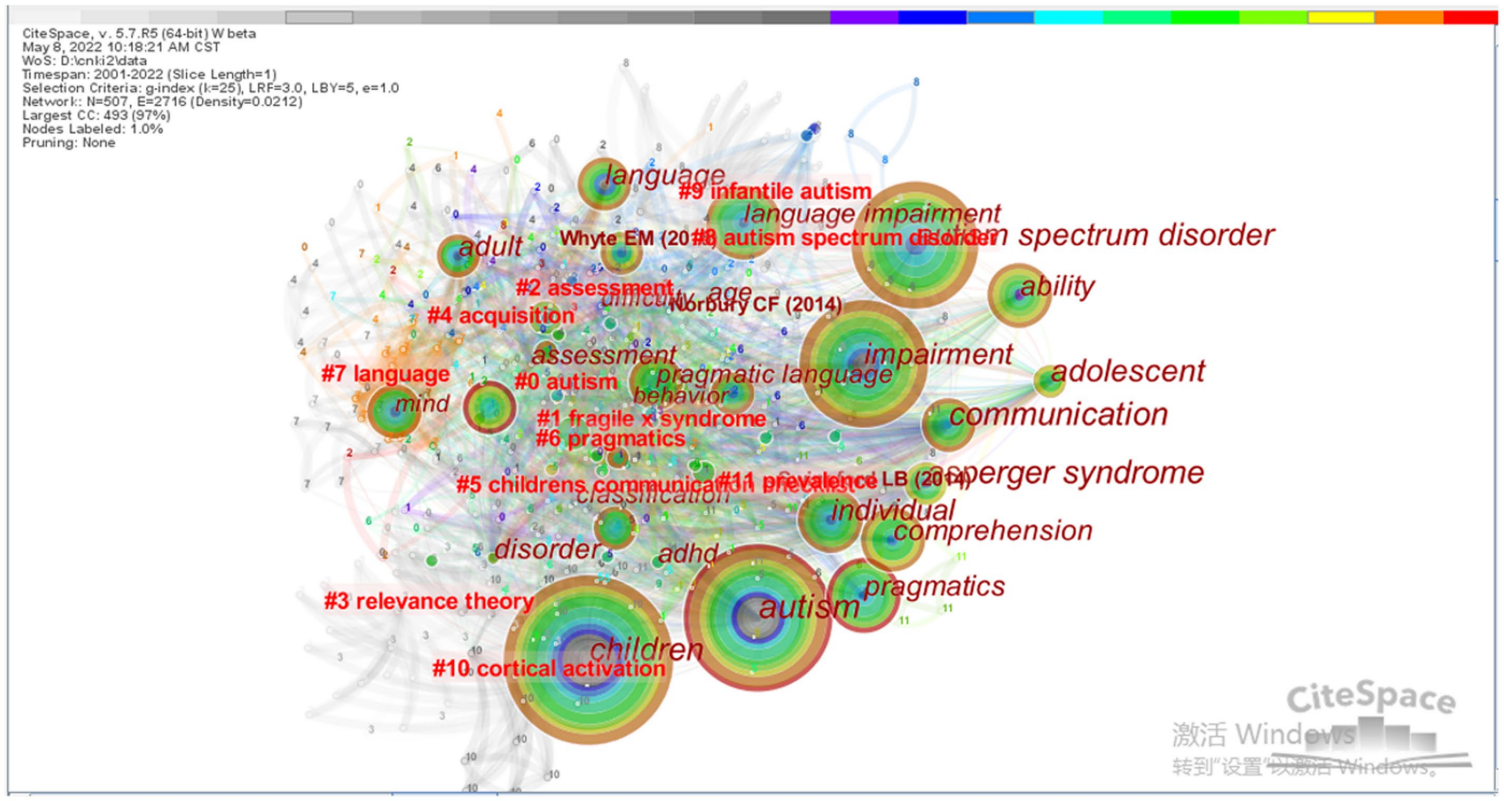
Clustering chart of key words in pragmatic impairment in ASD.

In the CiteSpace map, research hotspots are primarily identified by critically examining the high-frequency keywords embodied by the included studies. By mapping the co-occurrence and clusters of keywords, the knowledge landscape unveils the central domains of research focused on understanding the pragmatic impairment in autistic children (Figure 1). Researchers have directed attention toward speech comprehension, communication, cognitive processes, and other abilities that are intertwined with pragmatic impairment in ASD. These multifaceted studies highlight the complex nature of this condition and its impact on various aspects of an individual’s language development.

Furthermore, the dense connections among keywords demonstrate extensive and interdisciplinary research collaborations in the realm of pragmatic impairment in ASD. This collaborative approach has fostered numerous academic achievements, indicating a positive trend in the development of knowledge in this field. The geographical distribution of research on pragmatic impairment in ASD underscores the breadth of global cooperation and the closely connected coordination among researchers from diverse fields, enriching the overall understanding of the disorder and its implications. The comprehensive investigation of pragmatic impairment in ASD and its interplay with different domains underscores the significance of this research area. By integrating insights from various disciplines and fostering collaborative efforts, scholars have made significant strides in advancing knowledge and contributing to the well-being of individuals with ASD and related conditions.

It can be seen from the keyword clustering diagram of Figure 1 that 12 clusters have been identified in the research on pragmatic impairment in ASD from 2001 to 2022. These clusters are as follows: #0 autism, #1 Fragile X syndrome, #2 assessment, #3 relevance theory, #4 acquisition, #5 children communication, #6 pragmatics, #7 language, #8 autism spectrum disorder, #9 infantile autism, #10 cortical activation, and #11 prevalence.

Table 1 illustrates the statistics of high-frequency keywords. From the evidence of the keyword co-occurrence, keyword clustering (Figure 1), and the statistical findings presented in Table 1, the past two decades witness the following three main research aspects of pragmatic impairment in ASD.

**Table 1:** High frequency keyword and high centrality keyword ranking.

Order	High frequency keywords	Frequency	High-centrality keywords	Centrality
1	Autism	72	Communication	0.22
2	Communication	36	Autism	0.16
3	Asperger syndrome	28	Asperger syndrome	0.16
4	Disorder	18	Disorder	0.11
5	Intervention	7	Intervention	0.03
6	Pragmatic language impairment	6	Activation	0.02
7	Specific language impairment	5	Diagnostic observation schedule	0.01
8	Diagnostic observation schedule	5	Diagnosis	0.01
9	Diagnosis	4	Joint attention	0.01
10	Joint attention	4	Association	0.01
11	Association	4	Emotion	0.01
12	Emotion	3	Narrative	0.01
13	Narrative	3	Communicative development	0.01
14	Communicative development	3	Pragmatic language impairment	0.01
15	Activation	3	Specific language impairment	0.00

#### 3.1.1 Assessment and diagnosis of pragmatic impairment in ASD

The Screening and diagnosing of language impairments in autistic children has long been a research focus as it serves as the premise of the development of pragmatic intervention and their efficiency evaluation. Traditionally, the initial approach to ASD assessment was grounded from a phenomenological perspective, focusing on observable behavioral characteristics. However, assessing autistic children to identify their functional and categorical properties accurately is significantly challenging because of the intrinsic complexity of ASD. This complexity, when scoped at the language aspect, arises from the multifaceted impairments encompassing verbal and nonverbal communication, emotional regulation, cognitive function, and social development. The intricate nature of these concurrent impairments adds substantial complexity to the diagnostic process regarding language impairment among autistic children. Moreover, the etiology of ASD remains enigmatic, further complicating our understanding and approach to diagnosis and intervention.

The initial identification of pragmatic impairments in autistic children was documented by Bishop and Rosenbloom (1987) and Rapin and Allen (1983), who delineated the symptoms and critical values associated with this impairment. After numerous iterations, the Children’s Communication Checklist (CCC-1) was devised as a comprehensive tool to detect potential communication barriers and evaluate issues related to pragmatic competence, speech characteristics, social interactions, and interests in children. As a structured informal instrument (Bishop, 1998; Cummings, 2009), CCC-1 elucidates the interplay between pragmatic impairment and other language development concerns. It serves as a screening tool to distinguish between typically developing children, aged 4–7 years, and those demonstrating pragmatic language impairments within the same age bracket, particularly among autistic children (Volden and Phillips, 2010). CCC-1 employs a 132-point cutoff to differentiate children with Specific Language Impairment (SLI) from those experiencing pragmatic impairment.

Notably, the Pragmatic Composite Score (PCS), as the pragmatic component of the CCC-1, identifies pragmatic deficits in autistic children. PCS is computed by summing five pragmatic subscale scores: (A) inappropriate initiation, (B) coherence, (C) stereotyped conversation, (D) use of context, and (E) rapport (24:883). A lower PCS score denotes more pronounced pragmatic issues in autistic children. PCS has demonstrated efficacy in distinguishing autistic children from those without ASD (Bishop and Baird, 2007; Charman et al., 2007; Deckers et al., 2020). However, Bishop also noted the limitations of CCC-1, such as its excessive emphasis on a single source of the problem and its less satisfactory generalizability to be applied to children with pragmatic impairments across different countries, age groups, and types. To address these limitations, Bishop re-visioned CCC-1 in 2003 to CCC-2, providing quantitative evaluations of children with pragmatic impairments and enabling early screening for additional language assessments. Nonetheless, both the validity of CCC-1 and CCC-2 are primarily restricted within the children in the UK and the US.

Additionally, the Social Responsiveness Scale (SRS) (Constantino and Gruber, 2005), evolving into modified SRS-2 (Constantino and Gruber, 2012) is a widely used quantitative assessment tool designed to measure the social impairments associated with ASD. With several subscales of social awareness, social cognition and social communication, some items on the SRS may indirectly capture certain aspects of pragmatic difficulties of individuals between the ages of 2 years 6 months through adulthood. Furthermore, the Test of Pragmatic Language (TOPL) stands out as a specialized tool aimed at assessing the social use of language across various contexts. It is especially beneficial for evaluating individuals with conditions like ASD, where pragmatic difficulties are a common occurrence.

However, some scholars still have reservations about the effectiveness of using scales to measure this neuro-developmental disorder with a series of heterogeneous conditions. For example, researchers focused on CCC-2 also suggested that “it is unrealistic to use the CCC-2 to make categorical distinctions on this continuum of disorder (Norbury et al., 2004).” Although it is undeniable that an initial assessment of ASD could provide a relatively accurate appraisal of the individual’s difficulties and strengths, its lasting effect for autism symptom severity is uncertain. Further, different from SRS, a self-report scale, CCC-2 is a parent report instrument, which may be more subjective. Therefore, the combination of various scales targeting different aspects of pragmatic impairments of ASDs should be considered for utilization in future research to generate a more comprehensive description of the development of ASD individuals.

It’s important to note that while the scales, tests and checklists above are valuable tools for assessing pragmatic language, they are best used in conjunction with other assessments and observations to form a comprehensive understanding of an individual’s pragmatic language abilities. Pragmatic language skills are complex and can vary widely among autistic children, so a multi-faceted approach to assessment is often recommended. Accordingly, there is an ongoing need for a universally applicable tool that can accurately assess and diagnose pragmatic impairments in autistic children.

#### 3.1.2 Intervention strategies for pragmatic impairment in autistic children

The importance of early intervention for the pragmatic competence of autistic children has been evidenced by recent studies upon neuroplasticity (Courchesne et al., 2007, 2011; Desarkar and Rajji, 2015). With a special focus on interventions targeting pragmatic impairment in children with autism, it is noteworthy that while autism symptoms typically become apparent within the initial three years of life, formal diagnosis is often delayed until the age of 3 or 4 (Mandell et al., 2005). Nevertheless, there remains a viable possibility for detecting autism at an earlier stage (Landa, 2007). Early intervention strategies have demonstrated the potentials of promoting autistic children’s pragmatic competence embedded in their social development from an early age (Landa and Holman, 2005; Kasari et al., 2006). The experiences of social interactions have a profound impact on cortical specialization, which pertains to the fine-tuning of sensory systems and the enhancement of both intra-regional and inter-regional neural integration or connectivity, which are all critical elements in children’s early neurodevelopment. Enhanced neurofunctions from social interaction are anticipated to offer neurological backing for children’s growth, allowing them to demonstrate more intricate and adaptable behavioral patterns. In this case, early social interactional activities, typically enhanced by early pragmatic interventions, are supposed to promote greater cognitive flexibility and a broader range of pragmatic knowledge, enabling autistic children to exhibit more contextually appropriate behaviors in unfamiliar social situations for the purpose of improving pragmatic capabilities.

Recently, direct motor interventions have gained significant attention in research, particularly regarding the enhancement of the motor system’s development, the investigation into the intricacy of motor requirements in intervention activities, and the complexity and familiarity of the requisite actions related to sound, gestures, movement patterns, or imitation sequences within games. For instance, during song gesture games, autistic children are encouraged to move their arms rhythmically. The practices of mimicking others, sustaining attention, and responding in a reciprocal, meaningful, and contingent manner based on others’ behaviors have been empirically shown to boost communicative skills in autistic children. Such practices play a significant role in enhancing their pragmatic language abilities (Whalen and Schreibman, 2003; Ingersoll and Schreibman, 2006). Game-based interventions foster the pragmatic language skills of autistic children in an engaging, fun manner. Through these games, diverse social situations are simulated, encouraging children with autism to use appropriate language.

Furthermore, several studies focus on educational intervention for autistic children (Lord and McGee, 2001). The National Research Council of the National Academy of Sciences defines education for autistic individuals as a method to facilitate the acquisition of skills or knowledge, including adaptive skills, language and communication, socialization, academic learning, and the reduction of inappropriate behaviors. The aim is to maximally promote the independence of autistic children, improve their quality of life, and alleviate family stress (Myers and Johnson, 2007; Stahmer et al., 2011). When correctly implemented, these strategies have been shown to be effective for autistic children (Wong et al., 2015). Therefore, various behavioral intervention strategies have been attempted, such as breaking down desired behaviors into manageable tasks, and teaching them through a structured set of behaviors known as discrete trials, which comprise stimuli or antecedents, behavior, and consequences (A-B-C). Such interventions can help autistic children develop appropriate social skills, actively participate in communication with others, and use appropriate language, which serves as a great contributor to the pragmatic impairment of autistic children.

Importantly, empirical evidence underscores the efficacy of social communication interventions in mitigating pragmatic impairments in children with autism (Watkins et al., 2017; Ostryn and Mincic, 2022; Gabarron et al., 2023; Wattanawongwan et al., 2023). Techniques such as the utilization of social stories, augmentative and alternative communication (AAC) tools, and the strategic employment of social media have garnered recognition for their effectiveness in fostering social communication skills. These techniques capitalize on play, naturalistic environments, and a spectrum of sensory modalities—including visual, auditory, and tactile channels.

For instance, the act of recounting and disseminating social stories—characterized by delineating situations using straightforward, intuitive language and proffering apt responses and resolutions—equips children with autism with a clearer comprehension of specific social scenarios, thereby facilitating the exhibition of socially expected behaviors. The deployment of AAC tools, encompassing modalities like picture cards, symbolic systems, or augmented reality applications, empowers these children by offering avenues to articulate their thoughts and intentions. Such tools stand as robust pillars of support, catalyzing communication between children with autism and their interlocutors.

Furthermore, existing research advocates for the provision of social communication interventions within mainstream educational contexts, alongside neurotypical peers. Such an inclusive pedagogical approach is posited to accentuate social participation and enhance a range of communication competencies, inclusive of pragmatic language skills, in individuals with autism.

Currently, variables that impact the effects of intervention, such as participants’ age, the length of the intervention and the format of social stories, have been studied (Kokina and Kern, 2010). However, researchers have not yet conducted longitudinal studies on the timing of the effectiveness of such interventions and the duration of the intervention cycle. Considering that ASD has a long-term effect on individuals, the communication interventions that target ASD should be persisted in and future studies should take longitudinal research that centers on the duration of the intervention. The kind of thorough research is crucial for understanding how interventions affect the long-term recovery process of ASD individuals. Unfortunately, data in this field are still relatively scarce at present. Therefore, future research should focus on filling this knowledge gap to provide more reliable and targeted guidance for clinical practice.

In the broader context of therapeutic approaches, it is imperative to underscore the significance of evidence-based interventions targeting the enhancement of pragmatic language competencies in the social communication of children with autism. The variability in the autistic spectrum necessitates a nuanced approach; hence, intervention strategies ought to be meticulously crafted, taking into account the unique developmental, cognitive, and communicative profiles of each child. A one-size-fits-all methodology is not only suboptimal but could inadvertently exacerbate existing challenges. Thus, a comprehensive assessment, followed by individualized intervention plans, is paramount. Furthermore, the complexity of pragmatic language and its inextricable link to social cognition implies that such interventions will achieve optimal efficacy when executed in conjunction with professional medical oversight. Collaborative, interdisciplinary efforts that bridge the domains of speech therapy, cognitive neuroscience, and pediatric medicine can foster holistic improvements in the pragmatic language abilities of children with autism.

#### 3.1.3 Research on theory of mind in autistic children

Theory of Mind (ToM), as a branch of cognitive science, investigates mindreading or mentalizing or mentalistic abilities, which explains how we ascribe mental states to other persons and how we use the states to explain and predict the actions of those other persons (Smelser and Baltes, 2001). Difficulties in using language effectively in communicative exchanges, including understanding and using the intentions, context, non-literal and figurative meanings of speech may stem from a lack of (ToM). The deficits of ToM for autistic Children have challenges to understand others’ intentions, infer the meaning behind speech, and predict others’ behavior. For example, they may struggle with understanding and applying the social rules, reference, and metaphors of language (Baixauli-Fortea et al., 2019). Children with autism frequently face challenges related to ToM skills, notably in making inferences about the thoughts, beliefs, and emotions of others. These difficulties manifest in broader domains of social cognition and communication, thereby triggering their pragmatic impairments. Notably, research suggests a reciprocal relationship where such pragmatic impairments also contribute to their ToM deficits (Chevallier et al., 2011). As an illustration, children with autism often grapple with comprehending others’ beliefs and perspectives. This compromised understanding can, in turn, impede their ability to engage in fluid social interactions.

Instead of behavioral measures of implicit ToM in ASD (Deschrijver et al., 2016), newly promising neuroimaging technology, such as resting-state functional magnetic resonance imaging (rs-fMRI), has been used to assess the neural mechanism of ToM in ASD (Iosifyan et al., 2020). Notably, studies assessing cognitive aspects of ToM in ASD using techniques obtained in neuroscience is relatively rare. Hence, further studies should take this into consideration and delve more deeply into the neural mechanism of ASD. Also, although the use and effectiveness of therapeutic programs aimed at developing ToM in ASD have been confirmed, researchers pointed out that further assessments are still necessary (Dyrda et al., 2020).

Interestingly, it is important to note that ToM is a complex skill that develops gradually over time, and not all autistic children have the same pattern of impairments. And some may be particularly good at certain aspects of ToM such as recognizing emotions, while struggling with others like understanding intentions (Baixauli-Fortea et al., 2019). Studying upon ToM can help us understand the pragmatic impairment in autistic children, but we cannot simply attribute pragmatic impairment in autistic children to deficits in ToM. Autism is a complex neurodevelopmental disorder, and their specific manifestation of pragmatic impairment can vary among individuals, considering their broader symptomatology.

### 3.2 Research trend characteristics

**Figure 2:**
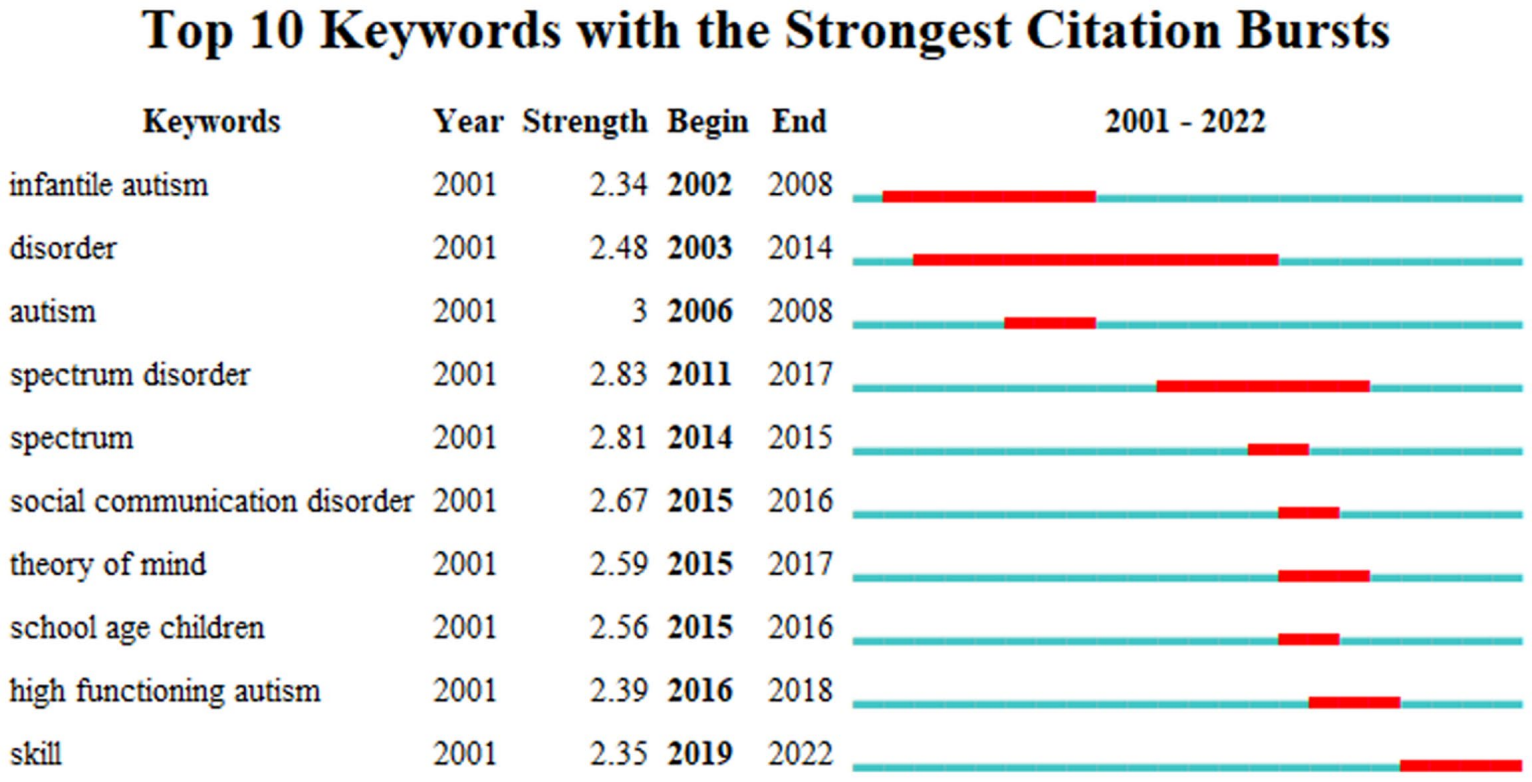
Analysis of keyword emergence in the study of pragmatic impairment in children with ASD.

A comprehensive analysis of research trends necessitates the identification of emerging keywords, which were collected carefully from every paper. Those keywords within papers, which are more detailed and profound than key terms we have employed to search the related papers, represent the cutting-edge areas of interest in a particular field during a specific time frame. As depicted in Figure 2, while the research emphasis may shift across different years, certain themes consistently emerge, forming the foundational concepts and enduring hotspots in the research pertaining to pragmatic impairment in autistic children.

From 2002 to 2017, the primary focus was on autism spectrum disorders in young children, with keywords such as “infantile autism,” “disorder,” “autism,” and “spectrum disorder” frequently appearing in the papers. Subsequently, in the period from 2015 to 2018, researchers pivoted their primary areas of investigation toward keywords like “high-functioning autism,” “school-age children,” social communication disorder, and “theory of mind.”

From 2019 to 2022, the keyword “skill” emerged as a prominent trend, indicating a future frontier and direction in research. This evolution in focus elucidates the progression of research on pragmatic impairment in children with autism: from early descriptions and interpretations of autism spectrum disorders in young children, to the examination of social interaction difficulties and theory of mind in high-functioning autistic school-age children, to the current focus on the investigation of their specific skills.

**Figure 3:**
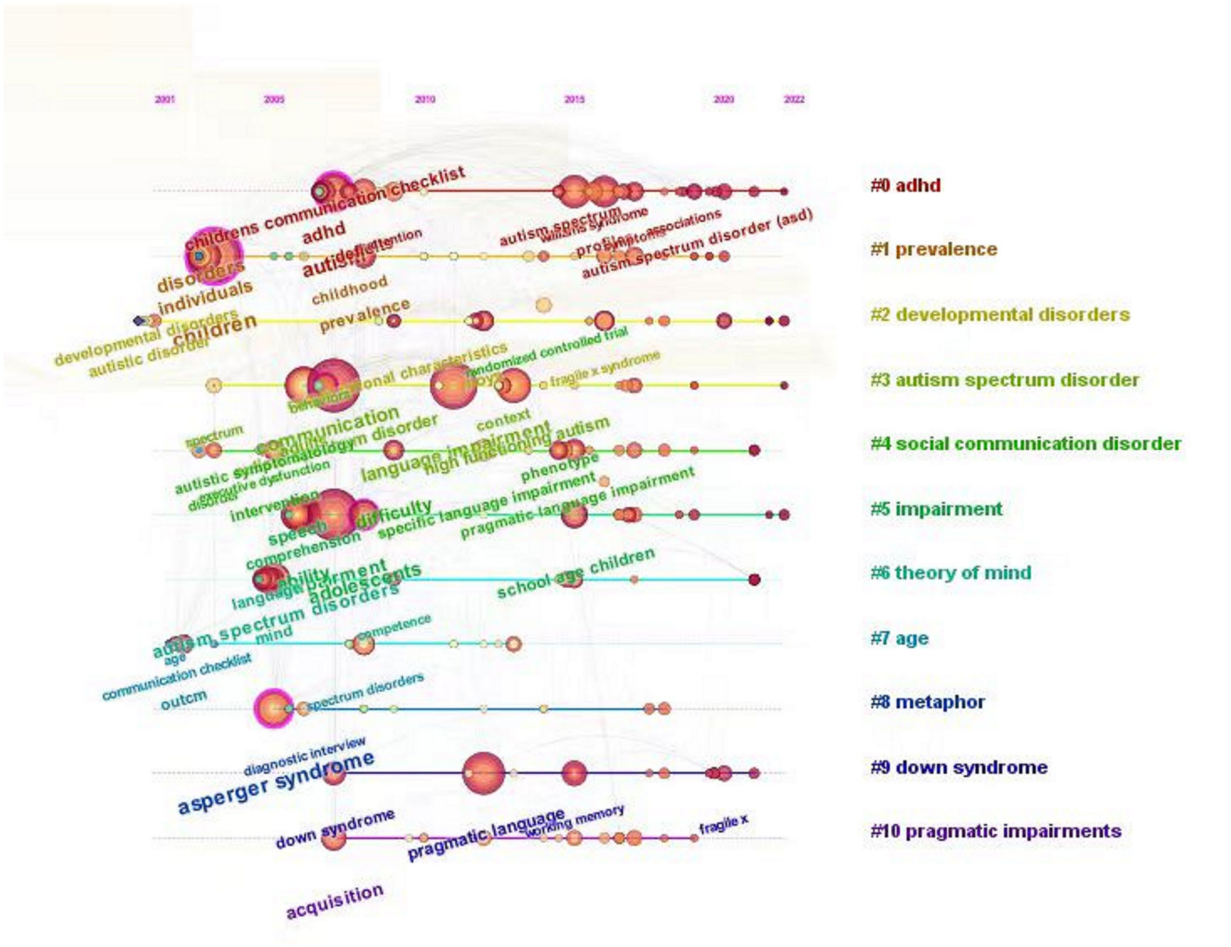
Keywords Time Chart in Pragmatic Impairment in ASD.

The central topic and content which aroused researchers’ interest have been presented in Figure 3. Specifically, the developmental disorders/social communication disorder have repeated twice, indicating that individuals with ASD are in dynamic developmental conditions and characterized by language deficits. The emergence of the term “age” indicates that autism undergoes continuous development and changes at different stages, affecting the lives of individuals over time. Therefore, we should focus on the manifestations of autism across different age groups, especially school-aged children with ASD. Meanwhile, “theory of mind” and “metaphor” demonstrated that the impairment of cognitive state could be a good source for the assessment of ASD. In addition, the conversation/communication checklist and intervention in the right part of Figure 3 suggested that the salient characteristics of ASD are dysfunctions of communication and the efficient interventions should target social interaction.

### 3.3 The evolution of research path

The timeline of keywords represents the temporal progression of academic hotspots in scientific research. With the progression of time, the increasing density of keywords and their extensive interconnections indicate an escalating interest and focus among researchers on the study of pragmatic impairments in autistic children. This leads to expanding and deepening of the research field regarding interconnectedness and depth of investigation. The keyword clustering clearly signifies the prevailing research directions. Apart from the overarching research focus on pragmatic impairment in autistic children, other significant research directions include #0 schizophrenia spectrum, #1 case study, #2 conversational gesture, #3 structural language, #4 irony comprehension, #5 pragmatic profile, #6 blind children, #7 mirror neuron dysfunction, and #8 longitudinal trajectories.

The analysis of the timeline of keywords in the research on pragmatic impairment in autistic children provides insights into the research’s basic context and evolutionary trajectory.

1. The period from 2001 to 2007 marked the early developmental stage of research in this field. Studies during this time concentrated on issues such as differential diagnosis of interactive social disorders (Scheeringa, 2001), language delay and autism characteristics (Michelotti et al., 2007), and representation and classification of language deficits (Rapin and Dunn, 2003). Some studies also explored the importance of interventions (Tager-Flusberg and Caronna, 2007). Among the research outputs, the article “EEG evidence for mirror neuron dysfunction in autism spectrum disorders” (Oberman et al., 2005), published at the 11th annual meeting of the Cognitive Neuroscience Society, provides scientific evidence for mirror neuron dysfunction through electroencephalography. It is the highest-cited literature in the researches on pragmatic impairment in autistic children and has had a significant impact.
2. From 2008 to 2014, there was a slow but steady increase in related research results. Researchers focused on the disorder representation of verbal and non-verbal factors, such as prosodic speech brainstem encoding deficits (Russo et al., 2008), prosodic testing experiments (Smelser and Baltes, 2001), specific deficits in language understanding (Gernsbacher and Pripas-Kapit, 2012), and gesture expression in conversation (Marchena and Eigsti, 2010). Concurrently, researchers explored the diagnosis of pragmatic impairment in autistic children from the perspectives of diagnostic classification methods for specific language disorders (Gibson et al., 2013), diagnostic interviews, and diagnostic observation tables (Leyfer et al., 2008). In addition, some researchers conducted comparative analyses of atypical language development in individuals at high clinical risk of schizophrenia and autism spectrum disorders (Solomon et al., 2011).
3. Research outputs sharply increased in 2015, 2016, and 2017, forming the first peak period. During this period, researchers focused on several dimensions of the pragmatic impairment of children with autism. These dimensions include (1) syntax and discourse perspectives, including narrative syntax and story structure (Peristeri et al., 2017) and discourse output (Song et al., 2017); (2) conversational cooperation ability perspectives, including conversational behavior development (Van Den Heuvel et al., 2017) and conversational cooperation ability (Obeid et al., 2016) and social pragmatic difficulties (Hahn et al., 2017); (3) social interaction perspectives, i.e., early social communication of children with specific language impairment (Mok et al., 2014) and peer relationships (Hwang et al., 2017); (4) perspectives on non-verbal information in communication, such as the impact of facial expressions on understanding indirect pragmatic expressions (Kalandadze et al., 2018).
4. Since 2018, research on pragmatic impairment in autistic children has entered a stage of steady development. The application of advanced technology has become a new focus of interest. Researchers have begun using meta-analysis methods to analyze metaphorical language data (Larkin et al., 2017) and genotyping technology to reveal the association between CNTNAP2 variants and autism (Shiota et al., 2021). Rehabilitation practices like parent–child interaction (Bush et al., 2021) and exercise therapy (Caliendo et al., 2021) have also received academic attention.

As of mid-May 2022, the number of published articles on pragmatic impairments in autistic children has already reached 8. These studies encompass various aspects, such as narrative discourse (Félix et al., 2022), pragmatic competence (Fussman and Mashal, 2021), pragmatic features (Diez-Itza et al., 2022), and the diagnostic accuracy of communication checklists (Aghaz et al., 2022). It is anticipated that with the continuation of theoretical research and experimental exploration of pragmatic impairments in autistic children, the publication output will grow significantly.

## 4 Research outlook

Indeed, the research on pragmatic impairments in autistic children has made significant strides over the past two decades, undergoing four historical stages: early inception, slow growth, rapid expansion, and steady development. Currently as a forefront area of research in autism spectrum disorder, the field still faces several pressing issues that warrant further investigation.

Firstly, existing research primarily focuses on three facets: assessing and diagnosing pragmatic impairment in autistic children, intervention strategies, and social communication impairments. These are mainly explored and verified from clinical medicine, pedagogy, and linguistics perspectives. This body of research inspires further multidisciplinary inquiry integrating fields such as psychology, cognitive neuroscience, philosophy, speech pathology, anatomy, rehabilitation medicine, and genetics, and statistical and experimental methods. Exploring interdisciplinary and cross-domain collaborative efforts—marrying language disorder therapy with medical pathogenesis tracing and melding empirical findings with clinical rehabilitation practices—can help probe the pragmatic mechanisms implicated in the language acquisition processes of autistic children. Additionally, conducting comparative studies across multiple disorders can yield profound insights into the inherent biological attributes of neurons.

Secondly, there is a dearth of a universally accepted scale for globally assessing and diagnosing pragmatic impairment in autistic children. For example, the effectiveness of assessments varied from one to another and some scales, such as CCC-2, are subjective since the autistic severity is reported by parents or teachers rather than by the individual with ASD. Establishing a scale that universally accepted would facilitate the screening of children with pragmatic impairment and provide a robust foundation for future intervention strategies. Researchers from diverse fields and academic backgrounds should bolster communication and collaboration. A comprehensive examination of cognitive, linguistic, social, behavioral, and psychological aspects involved in pragmatic impairment could lead to the development of standardized strategies and methods to assess and quantify pragmatic skills and the formation of a theoretical framework for evaluating the scale.

Thirdly, the effectiveness of interventions for social communication impairments in autistic children significantly determines their subsequent rehabilitation outcomes and quality of life. Although the substantial importance is attributed to intervention studies for autistic children, the interventions often focus on single approaches such as motor, educational, and behavioral interventions. However, there is a lack of comprehensive intervention strategies that holistically enhance the verbal and non-verbal behaviors of autistic children from a pragmatic perspective based on objective data. To address this, event-related potentials (ERPs), functional magnetic resonance imaging (fMRI), and other experimental techniques can be utilized to accurately identify the specific temporal intervals and phase characteristics of the biological responses corresponding to stimuli during pragmatic processing in autistic children. This can help pinpoint activity patterns in related brain regions. Furthermore, comprehensive localized intervention strategies can be proposed based on assessment results from scales evaluating pragmatic impairments in autistic children from language, communication, and culture perspectives.

This study portrayed the knowledge framework of research on pragmatic impairment in autistic children and predicted future research frontiers, which could benefit scholars’ research in this field. However, some limitations still need to be acknowledged. First, due to the limitation of the analytical tool, the literature was mainly collected from web of science, so the results might not be comprehensive enough. We will conduct a more comprehensive literature review involving multiple databases such as Pubmed, Embase, Scopus and Google Scholar in the future. Second, the clusters of keywords may be a little different from the keywords for diagnosing pragmatic impairments of ASD in the DSM-5 such as social-emotional reciprocity impairment (e.g., failure in turn-taking), abnormalities in nonverbal communication (e.g., failure in using eye contact), and impairment in relationship maintenance (e.g., failure in adapting to different social contexts). This discrepancy may be due to the fact that the co-occurrence and clustering of keywords in CiteSpace is realized by analyzing the quantity and relationships of keywords in the included studies (He et al., 2021), so keywords with relatively low frequency may not be fully presented in the clustering of this study. In this sense, future research can be conducted based on the core diagnosis of pragmatic disorders among ASD children in DSM-5.

## Author contributions

YL: Writing – original draft, Writing – review & editing. XT: Writing – original draft. HM: Visualization, Writing – review & editing. LC: Funding acquisition, Writing – original draft, Writing – review & editing. PW: Visualization, Writing – review & editing. YG: Visualization, Writing – review & editing.
